# “以建辅赛-以赛孵教-以教促学”闭环教学创新模式的构建与实践——以微等离子体-发射光谱仪器装置搭建及痕量元素分析实验为例

**DOI:** 10.3724/SP.J.1123.2025.04005

**Published:** 2025-10-08

**Authors:** Wanyi XUE, Di KE, Xiao ZHANG, Xiaodong ZHOU, Ruining YANG, Qingcen CHAO, Mingli CHEN, Hao MENG, Ting YANG

**Affiliations:** 东北大学理学院，辽宁 沈阳 110004; College of Sciences，Northeastern University，Shenyang 110004，China

**Keywords:** 实验教学, 教学模式创新, 仪器装置搭建, 介质阻挡放电, experimental teaching, innovation in teaching mode, construction of instrument devices, dielectric barrier discharge （DBD）

## Abstract

实验教学作为学生理论知识与实践能力之间的桥梁，能够深化学生对知识的理解，提升学生解决复杂问题的能力，为培养高素质的化学拔尖创新人才提供关键支撑。东北大学理学院化学实验教学中心在把握高等教育发展规律与创新人才成长规律的基础上，瞄准人才培养中存在的科研与教学壁垒难以贯通问题，提出“以建辅赛-以赛孵教-以教促学”闭环教学创新模式。该模式立足拔尖创新人才培养要求，充分发挥学科竞赛的实践反哺作用，打造“竞赛孵化→教学转化→能力反哺”动态反馈机制。本文以化学创新设计大赛获奖作品《微等离子体-发射光谱仪器装置搭建及痕量元素分析》为例验证该模式在传统实验教学上的突破，通过4学时的实验课程设计，学生自主搭建介质阻挡放电微型原子发射光谱装置，结合氢化物发生进样技术完成痕量砷检测，在20~500 μg/L范围内呈现良好的线性关系，决定系数（*R*^2^）为0.997。教学实践表明，学生在动手搭建的过程中实现了对原理认知的深化和仪器构造的透明化理解，促进了教学质量的提升，为“两性一度”课程改革提供了可推广的创新范式。

## Abstract (en)

Experimental teaching， serving as a bridge between theoretical knowledge and practical competence， can deepen students’ understanding of theoretical concepts， and enhance their ability to solve complex problems， thereby playing a pivotal role in cultivating elite chemistry talents for the national innovation system. By understanding the laws of higher education development and innovative talent growth， the Chemistry Experiment Teaching Center of the College of Sciences at Northeastern University has innovatively proposed a closed-loop teaching model： “Construction Supports Competitions-Competitions Nurture Teaching-Teaching Promotes Learning”， aiming at the problem of difficult barrier between scientific research and pedagogy in talent development. This model is based on the requirements for cultivating top innovative talents， leveraging the practical feedback of disciplinary competitions， and creating a dynamic feedback mechanism of “Competition Incubation→Teaching Transformation→Competency Feedback”. Taking the award-winning project “Micro-Plasma Emission Spectrometer Setup and Trace Element Analysis Experiment” from the *Chemistry Innovation Design Competition* as an example， this study demonstrates the model’s breakthrough in traditional experimental teaching. Through a 4-class-hour experimental course， students independently construct a dielectric barrier discharge （DBD） micro atomic emission spectroscopy device， combined with hydride generation （HG） introduction technology to complete trace arsenic detection. The method showed a good linear relationship in the mass concentration range of 20-500 μg/L， with a coefficient of determination （*R*^2^） of 0.997. Teaching practice shows that students deepened their understanding of principles and made the instrument structure transparent through hands-on construction， promoting the improvement of teaching quality and providing a scalable innovative paradigm for the “high-order， innovative， and challenging” curriculum reform.

本文为“全国大学生化学实验创新设计大赛专辑”稿件。

本科教学是高校人才培养的核心环节，其质量紧密关系着高校竞争力提升^［1-3］^。化学作为实验学科，实验教学对高素质化学人才培养至关重要^［4］^。依据《教育部关于一流本科课程建设的实施意见》（教高函〔2019〕8号）提出的“两性一度”要求（即高阶性、创新性、挑战度），各高校正积极推进科研成果向实验教学转化，以替代传统验证性实验^［5，6］^。然而转化过程中普遍存在课程设计系统性不足、实操环节与教学需求脱节等问题，导致学生学习适应不足，难以实现科学“增负”目标。

东北大学理学院化学实验教学中心提出“以建辅赛-以赛孵教-以教促学”的闭环教学创新模式（图1），通过构建完善的实验平台，为学生创造参与学科竞赛的条件；再将优秀竞赛项目转化成实验教学内容，更新实验项目，促进教学改革发展；最后通过实验教学拓宽学生学术认知广度，提升其专业素养。该模式形成了一个从“平台构建-设施建设-教学实践-能力提升”的教育闭环，为教学内容更新注入了源头活水。学生通过实验竞赛亲自“打造”的实验项目，不仅解决了传统实验项目创新性不足、内容陈旧等问题，同时相较于教师科研成果的直接转化，又兼顾优化了教学实验项目的实操性和连贯性，打破了科研成果和实验教学之间的“壁垒”。同时，持续增加和改进的教学项目解决了传统实验项目简单、重复的问题，培养了学生的创新能力，形成了“竞赛孵化→教学转化→能力反哺”的动态循环。本文以在第三届全国大学生化学创新设计大赛“微瑞杯”东北赛区获得二等奖的作品《微等离子体-发射光谱仪器装置搭建及痕量元素分析》为例，阐述由学生竞赛项目转化为实验教学内容的实际案例。

**图1:**
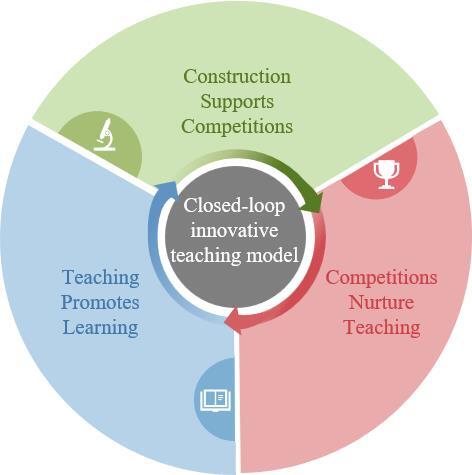
“以建辅赛-以赛孵教-以教促学”的闭环创新教学模式图

原子发射光谱法（optical emission spectroscopy，OES）具有选择性好、灵敏度高、分析速度快等优点，在化学^［7］^、冶金^［8］^、生命科学^［9］^、环境科学^［10］^等领域应用广泛，是仪器分析实验教学中必须掌握的一个重要检测方法。本科教学开设的原子发射光谱实验通常采用电感耦合等离子体光源作为激发光源^［11，12］^，虽然性能优异，但也存在如下问题：（1）仪器价格昂贵且规模较大，受空间和资源的限制，教学仪器设备台套数设置不足；实验教学通常为教师讲授原理、演示实验、学生进行简单的溶液配制和进样操作；一般情况为多个学生一组，难以确保每个学生都能进行完整的仪器操作。（2）商品化的原子发射光谱仪器无法拆解演示，学生通常只是按部就班地在电脑上进行参数设置、进样、换样、分析结果。仪器内部对于学生而言是难以捉摸的“黑盒子”，学生无法深入了解原子发射光谱的仪器构造及测定原理，教学效果难以保证。

我们将《微等离子体-发射光谱仪器装置搭建及痕量元素分析》这一获奖作品转化成4学时的教学实验项目，引入介质阻挡放电（dielectric barrier discharge， DBD）-微型原子发射光谱系统的研究成果，让学生自主搭建基于氢化物发生（hydride generation，HG）进样和微等离子体激发源的微型原子发射光谱仪器装置。本实验所需元件成本较低、实验装置小巧且简便、检测性能较好，适用于本科教学实验推广。在设备搭建过程中，学生不仅增强了动手操作与创新设计能力，还增强了学习和科研兴趣；同时，该实验打开了仪器设备的“黑盒子”，加深了其对原子发射光谱仪器构造及工作原理的认识和理解。

## 1 实验部分

### 1.1 实验原理

介质阻挡放电是指在电极之间插入介质阻挡层（通常是陶瓷、石英、聚四氟乙烯和玻璃等），阻挡两电极间的气体在高频高压条件下被击穿，产生微等离子体的现象^［13，14］^。本实验采用氢化物发生进样方式，结合介质阻挡放电-微等离子体原子发射法对痕量元素进行定量分析。如图2所示，样品和还原剂通过蠕动泵同时进入混合器内发生如下反应：3BH_4_^-^+4AsO_2_^-^+H_2_O+7H^+^=3H_3_BO_3_+4AsH_3_↑。随后进入气液分离器，废液从下方通过蠕动泵排出，反应产生的AsH_3_气体则由氩气从上方带出，通过干燥管除去水蒸气后进入石英管，两电极间接通适当高频电压进行介质阻挡放电，产生微等离子体，激发AsH_3_产生原子发射，通过电荷耦合器件（charge coupled device，CCD）光纤光谱仪采集所获得的原子发射光谱信号，从而构建微型原子发射光谱仪器装置。

**图2:**
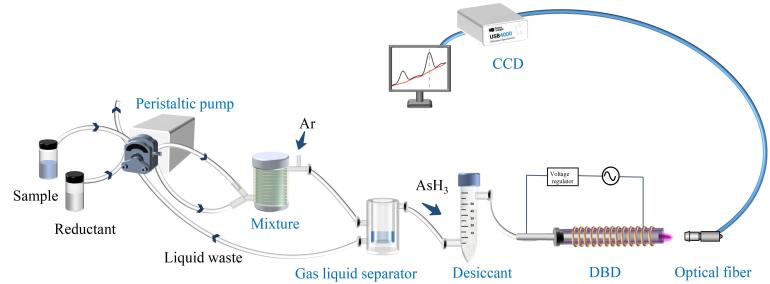
实验原理图 DBD： dielectric barrier discharge； CCD： charge coupled device.

### 1.2 仪器与试剂

仪器：BT100M蠕动泵（保定准择恒流泵制造有限公司）；FL-802气体质量流量控制器（深圳弗罗迈测控系统有限公司）；USB4000光纤光谱仪（美国海洋光学公司）；HB-C06电子霓虹灯电源（佛山南海虹霸电子有限公司）；TDGC2-0.5kVA接触调压器（浙江德力西电气有限公司）；QP400-2-SR石英光纤（美国海洋光学公司）。

其他实验耗材：气液分离器、石英管（内径1 mm，外径2 mm，长50 mm）、铜丝、生料带、热熔胶枪、蠕动泵管等。

试剂：亚砷酸钠标准溶液（NaAsO_2_，0.100 mol/L，北京北方伟业计量技术研究院）；盐酸（HCl，优级纯，国药集团化学试剂有限公司）；氢氧化钠（NaOH，优级纯，天津市大茂化学试剂厂）；硼氢化钠（NaBH_4_，98%，国药集团化学试剂有限公司）；抗坏血酸（C_6_H_8_O_6_，分析纯，天津奥普升化工有限公司）；硫脲（CH_4_N_2_S，分析纯，天津市北联精细化学品开发有限公司）；无水氯化钙（CaCl_2_，分析纯，天津市大茂化学试剂厂）。

### 1.3 实验步骤

#### 1.3.1 As标准溶液和还原剂的配制

盐酸是参与HG的重要部分，影响着HG效率和产生的H_2_量。适量的H_2_与工作气体Ar混合形成连续且稳定的等离子体，在激发能力和降低光谱背景之间取得平衡，进一步影响放电微等离子体的强度和稳定性^［15］^；另外，由于蒸气产生的效率受元素氧化态的影响，在实验中使用抗坏血酸和硫脲的混合物防止As（Ⅲ）氧化为As（Ⅴ）。我们向100 mL质量分数为5%的盐酸溶液中加入1 g硫脲和1 g抗坏血酸，以此为溶剂逐级稀释As储备液，得到质量浓度分别为20、50、100、200、300、400、500 μg/L的As标准溶液。称取0.5 g NaBH_4_溶于含有0.5 g NaOH的100 mL水溶液中，混匀得到还原剂，溶液需要现用现配。

#### 1.3.2 微等离子体原子发射光谱仪器的搭建

制作DBD装置 将不锈钢管一端插入石英管内约15 mm，使不锈钢管和石英管形成同轴圆柱形结构，接口部分用生料带固定，防止装置漏气。在石英管外壁紧密缠绕并固定铜丝。不锈钢管和铜丝分别与高频高压电源相连，由触控调压器调节输出电压。

搭建微等离子体原子发射光谱仪 将蠕动泵、混合器、气液分离器、干燥管、DBD装置、CCD光谱仪依次连接好。将蠕动泵管1、2、3分别安装到蠕动泵3个通道上，泵管1和2经过蠕动泵将待测样品溶液和还原剂运输到混合器，反应后进入到气液分离器，然后Ar将产生的氢化物气体经干燥管引入到DBD装置，液体废物从气液分离器下端由泵管3排出。高频高压电源输出端分别与不锈钢管和铜丝连接，提供高频高压从而产生稳定的等离子体。调节光纤探头的位置，确保CCD光谱仪高效采集DBD所产生的发射光谱信号，图3为教学过程中搭建好的微等离子体原子发射光谱仪。将装置连接好后，检查装置是否漏气。

**图3:**
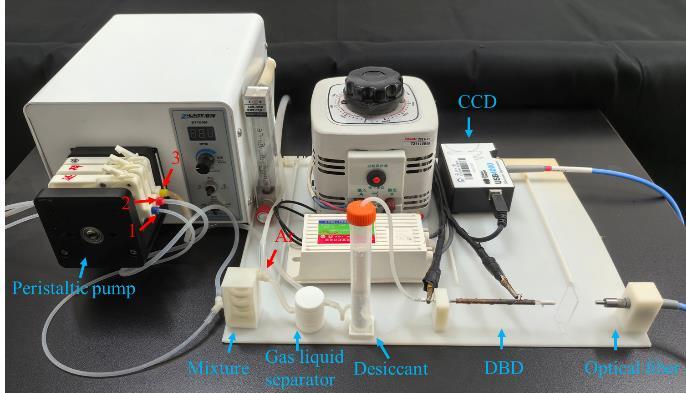
微等离子体原子发射光谱装置图

As元素分析 开启蠕动泵调整转速40 r/min，将As样品溶液和还原剂溶液混合，产生的AsH_3_气体经气液分离器由氩气引入DBD中激发，获得的特征发射光谱由CCD光谱仪记录。通过标准曲线法对As样品溶液中的痕量元素进行分析。

## 2 结果与讨论

### 2.1 可行性验证

为了考察HG-DBD-OES方法测定As的可行性，在输入电压为50 V的条件下，我们用500 μg/L的As标准溶液和相同条件下不含As的空白溶液进行对比。实验结果如图4所示，在引入空白溶液和还原剂NaBH_4_时，氢化物发生产生H_2_，谱线主要包括OH（309 nm）、NH（337 nm和357 nm）等，这些发射峰是由Ar-H_2_以及实验过程中掺杂的少量空气引起的背景发射光谱^［16］^；在引入As标准溶液后，可以观察到5条来自As的原子发射特征谱线（228.60、234.75、273.86、277.33和285.37 nm），由此可以证明本方法的可行性。本实验选择强度较高的234.75 nm作为As的特征谱线。

**图4:**
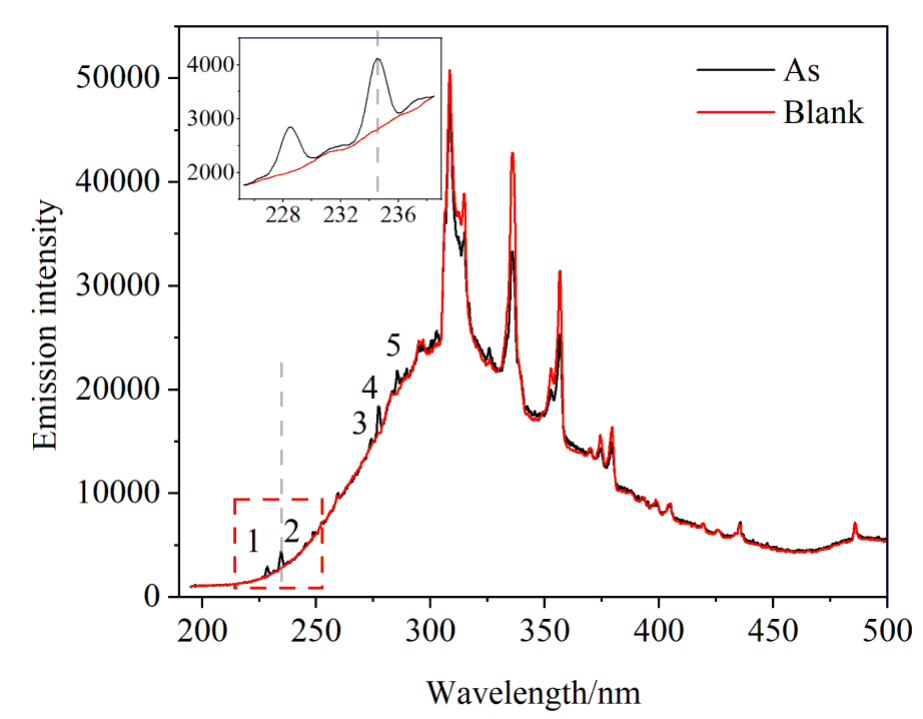
Ar-H_2_背景发射谱图和As的HG-DBD-OES发射谱图 HG： hydride generation； OES： optical emission spectroscopy. The atomic emission characteristic lines of arsenic （As） are as follows： 1. 228.60 nm； 2. 234.75 nm； 3. 273.86 nm； 4. 277.33 nm； 5. 285.37 nm.

### 2.2 条件优化

#### 2.2.1 硼氢化钠浓度

我们以As在234.75 nm处的发射峰为特征线，以233.21 nm为校准线，以相对发射强度进行条件优化实验。首先，氢化物发生反应中还原剂的浓度对分析性能至关重要，在本实验中NaBH_4_的量影响着AsH_3_的生成效率。实验优化了质量分数在0.1%到1.0%范围内的NaBH_4_溶液对As相对发射强度的影响，结果如图5所示。当NaBH_4_质量分数为0.5%时，As的相对发射强度达到最大值。NaBH_4_继续增加后，反应产生的H_2_共生量增加，一方面影响DBD微等离子体，一方面稀释了气态待测物，导致了As相对发射强度的降低。因此本实验采用质量分数为0.5%的NaBH_4_溶液为还原剂。

**图5:**
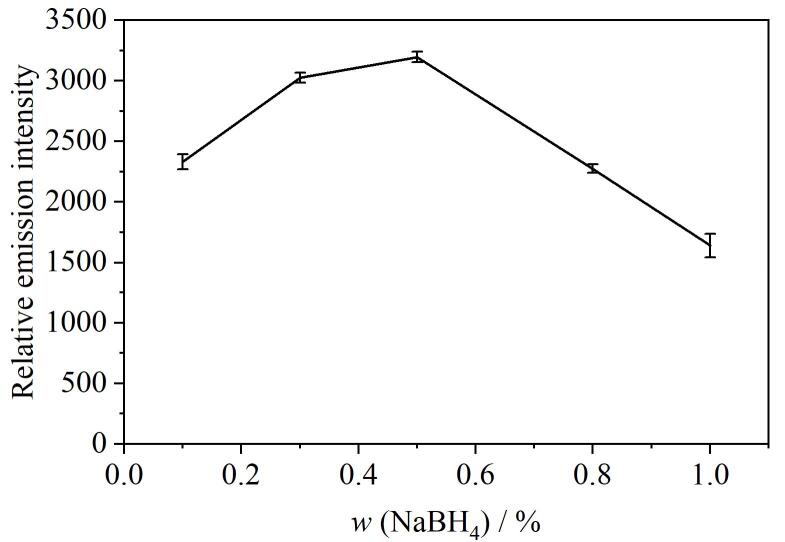
NaBH_4_质量分数对As相对发射光谱强度的影响（*n*=3）

#### 2.2.2 氩气流速

我们使用氩气为DBD反应的工作气体，氩气在装置中主要起到两个作用：（1）氩气通过产生高能量的亚稳态物质为激发As提供能量，是DBD反应中产生低温等离子体激发光源所必需的介质^［17］^；（2）氩气在氢化物发生过程中将AsH_3_气体经过气液分离器带入DBD装置中进行激发，进而完成测定，起到运输待测物的作用。本实验考察了氩气流速在0.1~0.5 L/min时对As相对发射强度的影响，如图6所示，氩气流速为0.4 L/min时，As相对发射强度最高。当氩气流速过低时，微等离子体激发能量低；当氩气流速过高时，AsH_3_被载气稀释，且在激发室停留时间短，因此氩气流速过低或过高都会造成信号的降低，本实验选择0.4 L/min时为氩气流速。

**图6:**
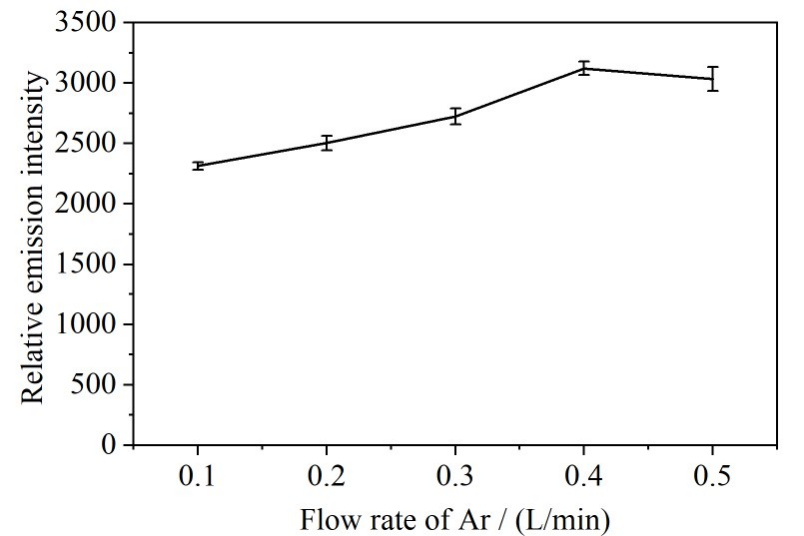
氩气流速对As相对发射光谱强度的影响（*n*=3）

#### 2.2.3 电压

在DBD装置的内外电极施加交流电压，为产生微等离子体提供能量。通过图7可知，在输入电压大于40 V时，即可产生微等离子体。随着输入电压增大，微等离子体激发能力增强，因此在40~125 V的范围内，As相对发射强度随着电压增大而增强。当电压超过100 V后，放电不稳定且容易击穿介质阻挡层的石英管，长时间放电影响电极寿命，因此本实验选择输入电压75 V为激发电压。

**图7:**
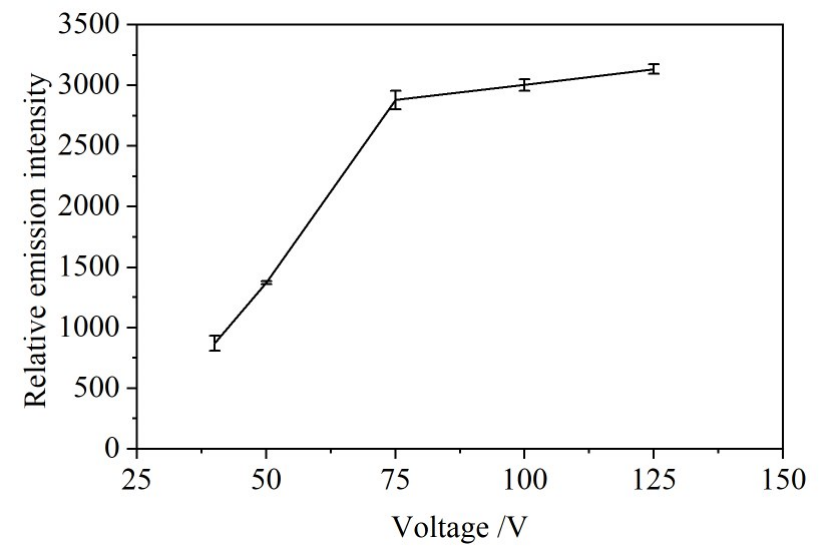
激发电压对As相对发射光谱强度的影响（*n*=3）

实验优化后的具体参数设置见表1，采用该实验条件时，实验教学可获得最佳效果。

**表1:** 教学实验参数设置

Parameter	Value
CCD spectrometer integration time	100 ms
Input voltage	75 V （AC）
Argon flow rate	0.4 L/min
NaBH_4_ mass fraction	0.5%
HCl mass fraction	5%

### 2.3 分析性能

我们对微等离子体-发射光谱仪器装置检测砷的性能进行了分析。

检测了一系列质量浓度的As标准溶液，结果表明，在20~500 μg/L范围内，As相对发射强度（*y*）与质量浓度（*x*）呈线性关系，线性方程为*y*=6.45*x*+1248.64，决定系数为0.997，拟合情况较好。

检出限（LOD）采用空白标准偏差法进行计算，计算公式为LOD=3*σ*/*S*，其中*σ*为11次空白样品测量值的标准偏差，*S*为通过线性回归分析获得的标准曲线斜率，测得检出限为17.24 μg/L。

以上结果能够满足本科实验教学的需求。

## 3 教学安排与反馈

### 3.1 实验教学安排

本实验在实施过程中设置为4个学时，具体安排见表2。学生分为2~3人一组，通过自主搭建小型微等离子体-发射光谱仪了解介质阻挡放电-微等离子体激发源的特点，掌握原子发射光谱的痕量元素分析方法。实验课程结束后，鼓励实验小组利用搭建的仪器装置进行拓展创新，开展更多元素的测试。本实验设置在应用化学专业专门化实验课程中，在2023年和2024年连续开展。

**表2:** 实验教学安排

Teaching stage	Class hours	Core tasks	Competency objectives
Safety education	0.5	safety protocols education and emergency training	awareness of safety protocols
Experimental principles	0.5	characteristics of DBD microplasma excitation source； principles of HG and its sampling advantages	understanding of device principles
Device construction	2	assembly of DBD reactor and circuit connection； integration and debugging of gas path system	precision operation； fault diagnosis
Performance testing	1	data acquisition and standard curve establishment	data analysis skills
After-class expansion	-	exploration of testing methods for new elements	innovative thinking cultivation

### 3.2 教学反馈与展望

本实验搭建的微等离子体-发射光谱仪成本低、装置小、检测性能较好，自主搭建仪器设备的教学设计使“黑箱操作”变为“透明建构”，非常适用于原子发射光谱法的实验教学。经过2年的教学实践，学生普遍认为，在自己动手组装与参数调试中加深了对原子发射光谱原理的理解，激发了对仪器探究的兴趣。同时提高了实验的参与度，培养了学生的动手能力和创新思维，促进了教学质量的提升。

目前，实验采用砷元素作为检测对象虽能达到教学目的，但其毒性存在一定安全隐患。我们将持续优化实验，在未来引入例如铋、硒等更绿色、安全的元素作为检测对象，二者适用于HG反应且毒性低于砷。同时铋元素检测可应用于胃药质控分析，硒检测可关联富硒农产品质控分析，能在降低安全风险的同时增强实验教学的实际应用性。

## 4 结语

“以建辅赛-以赛孵教-以教促学”的闭环教学创新模式具有自组织迭代、精准化适配、可持续增益的特点，通过构建“支撑学生竞赛-竞赛反哺教学-教学培养能力”的动态循环，打破了科研成果与教学应用的转化壁垒，突破传统实验教学单向知识传递模式，形成持续更新的教学实验项目自生长机制。展望未来，随着人工智能技术在教育领域的深入应用，我们将立足“以建辅赛-以赛孵教-以教促学”教学模式，通过融入虚拟仿真、3D打印等前沿技术，有效降低项目孵化的试错成本，提高个性化指导水平，从而进一步推进实验教学智能化转型。
